# Dermoscopy of rosacea: A cross-sectional study comparing dark and light phototypes

**DOI:** 10.1016/j.jdin.2025.05.022

**Published:** 2025-08-28

**Authors:** Kenza Khachani, Marwa Asermouh, Rasha Moumna, Asmae Yeznasni, Laila Benzekri, Karima Senouci, Mariame Meziane

**Affiliations:** a Department of Dermatology, Mohammed V University in Rabat, Ibn Sina University Hospital, Rabat, Morocco; b Department of Community Medicine Laboratory (Public Health, Preventive Medicine, Hygiene), Mohammed V University in Rabat, Ibn Sina University Hospital, Rabat, Morocco

*To the Editor:* Rosacea is a chronic inflammatory skin condition that is well-documented in light phototypes^1^^,^^2^ and less frequently reported in individuals with darker skin.^3^^,^^4^ Dermoscopy can be useful for the detection of the early features of rosacea that may not be visible clinically in dark phototypes.

Our study aimed to describe dermoscopic features of rosacea in dark phototypes and compared them with those in light phototypes.

This cross-sectional, descriptive, and analytic study included 206 patients with a clinical diagnosis of rosacea, confirmed or not by histopathology. It was conducted over 4 years at Ibn Sina University Hospital in Rabat. We divided patients into 2 groups: light phototypes (Fitzpatrick I, II, III) and dark phototypes ones (Fitzpatrick IV, V, VI). The dermoscopic variables were selected from the terminology of the International Dermoscopy Society Consensus. A *P*-value of <.05 was considered statistically significant.

The mean age was 45.8 years old, and women were predominant (86.9%) with a sex ratio (F/M) of 6.63. Dark phototypes comprised 68.4% of the sample.

We compared the 2 groups, and we found that the mean age was significantly higher in dark phototypes (*P* < .001). In both phototypes, the erythematotelangiectatic subtype was the predominant phenotype, followed by the papulopustular and the phymatous ones. The phototype was significantly associated with the erythematotelangiectatic (*P* < .001) and the papulopustular (*P* < .001) subtypes (Fig 1).

**Fig 1:**
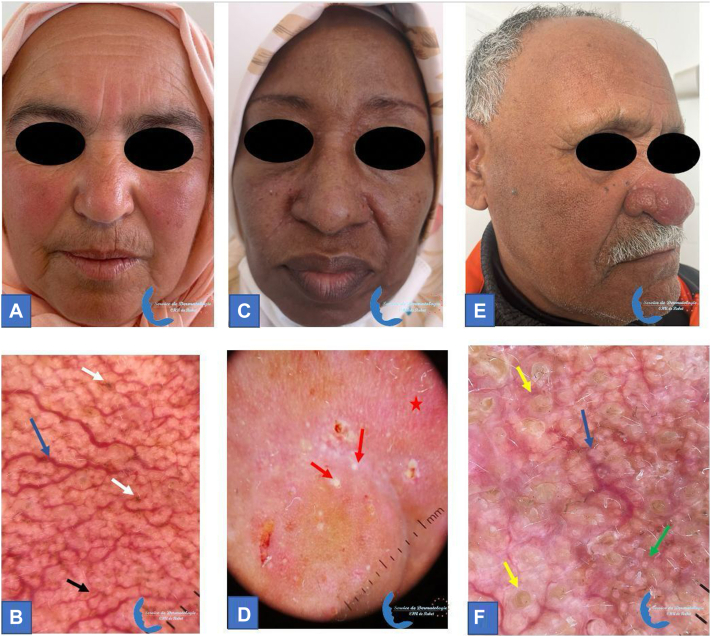
Rosacea clinical subtypes and their dermoscopic images in dark phototypes: erythematotelangiectatic subtype: **(A)** erythema on the cheeks and the forehead, **(B)** linear branched vessels (*blue arrow*) forming polygonal vessels (*black arrow*) in a reticular distribution, pigmented areas (*white arrow*); papulopustular subtype: **(C)** pustules on the cheeks and the nose, **(D)** pustules (*red arrow*), erythematous background (*red star*), telangiectasias; phymatous subtype: **(E)** ehinophyma, **(F)** follicular *yellow* clods (*yellow arrow*), rosettes (*green arrow*), and linear branched vessels (*blue arrow*).

Concerning dermoscopy, some features were significantly associated with the phototype: pigmented areas were predominantly seen in dark phototypes (*P* = .003), and orange structureless areas (*P* = .027) and crusts (*P* = .035) were predominantly seen in light ones (Fig 2). Other features were not associated with the phototype, such as vascular patterns (Figs 1 and 2), erythematous background, scales and follicular patterns (Table I).

**Fig 2:**
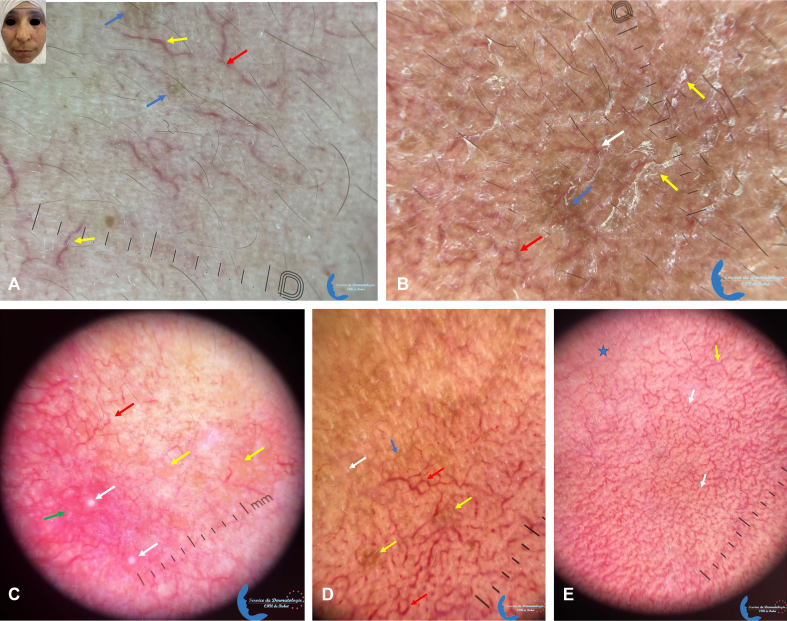
Dermoscopic features of erythematotelangiectatic rosacea in dark phototype: **(A)***blue arrow*: pigmented areas, *red arrow*: linear branched vessels, *yellow arrow*: linear curved vessels; **(B)***red arrow*: polygonal vessel, *yellow arrow*: scales, *blue arrow*: pigmented area, *white arrow*: linear branched vessels. **C,** Dermoscopic features of granulomatous rosacea in dark phototype: *white arrows*: pustules, *yellow arrows*: orange structureless areas, *green arrow*: erythema, *red arrow*: linear branched vessels. Dermoscopic features of erythematotelangiectatic rosacea in dark phototype: **(D)***red arrows*: polygonal vessels, *yellow arrows*: pigmented areas, *white arrow*: demodex tail, *blue arrow*: linear branched vessel; **(E)***white arrow*: polygonal vessels, *yellow arrow*: linear branched vessels, blue star: erythema.

**Table I:** Dermoscopic features of rosacea in dark and light phototypes

Dermoscopic features	Dark phototype	Light phototype	P
Pigmented areas	54.6% (*n* = 77)	32.3% (*n* = 21)	**.003**
Orange structureless areas	3.5% (*n* = 5)	12.3% (*n* = 8)	**.027**
Crusts	0.7% (*n* = 1)	6.2% (*n* = 4)	**.035**
Erythema	95.7% (*n* = 135)	92.3% (*n* = 60)	.308
Dotted vessels	5% (*n* = 7)	7.7% (*n* = 5)	.524
Linear vessels	44.7% (*n* = 63)	56.9% (*n* = 37)	.102
Linear branched vessels	61.7% (*n* = 87)	50.8% (*n* = 33)	.139
Linear curved vessels	21.3% (*n* = 30)	10.8% (*n* = 7)	.068
Polygonal vessels	66.7% (*n* = 94)	63.1% (*n* = 41)	.614
Comma like vessels	68.9% (*n* = 62)	43.1% (*n* = 28)	.904
Hairpin vessels	3.5% (*n* = 5)	6.2% (*n* = 4)	.468
Perifollicular pigmentation	12.1% (*n* = 17)	15.4% (*n* = 10)	.511
White scales	36.9% (*n* = 52)	32.3% (*n* = 21)	.524
Demodex tails	5.7% (*n* = 8)	4.6% (*n* = 3)	.754
Pustules	18.4% (*n* = 26)	23.1% (*n* = 15)	.439

Bold indicates that these features are the one significantly associated with the phototypes.

Dermoscopy showed features non-visible clinically. In 39% of dark phototypes, erythema was undetectable clinically but vascular patterns and pigmented areas were seen with dermoscopy. Pustules were observed in 18.4% of dark and 23.1% of light phototypes. We found that 13.8% of dark phototypes presented pustules inapparent clinically with a significant association (*P* < .001).

Pathogenesis of rosacea involves skin barrier and vascular alterations.^5^ This leads to more superficial blood vessels and scales that are seen with dermoscopy. In all of the studies, vascular patterns were predominant.^1^^,^^2^^,^^4^^,^^5^ Some particularities were seen in dark phototypes such as perifollicular pigmentation.^4^ In our study, pigmented areas were associated with dark phototypes because of the pigment lability and the darker background. This feature may result from vascular changes and post-inflammatory hyperpigmentation. While not specific, this pigmentation may help in the retrospective diagnosis of rosacea in dark phototypes.

The darker background can mask some features (erythema, pustules, and vascular structures). This explains why dark phototypes were older in our study. We demonstrated that pustules and vascular patterns are better seen with the dermoscope. Limitations include the monocentric design and the inclusion of patients undergoing treatment.

In conclusion, we highlighted dermoscopic features of rosacea in dark and light phototypes and the difference between them. Our study has the largest sample of patients and includes both phototypes, and all of the subtypes. Dermoscopy enhances recognition of rosacea subtypes by detecting pustules, pigmentation, and vessels. In the papulopustular subtype, dermoscopy can reveal non-visible pustules, guiding the treatment with cyclines.

## Conflicts of interest

None disclosed.
